# Patients’ knowledge and attitudes to the Wise List - a drug formulary from the Stockholm Drug and Therapeutic committee

**DOI:** 10.1186/s12913-018-2968-2

**Published:** 2018-03-12

**Authors:** Pia Bastholm-Rahmner, Lars L. Gustafsson, Kristina Aggefors, Kristina Ateva, Susanne Elfving, Jaran Eriksen, Malena Jirlow, Maria Juhasz-Haverinen, Rickard E. Malmström, Mahan Nikpour-Ardaly, Magnus Röjvall, Martina Vallin, Eva Andersén-Karlsson, Marie-Louise Ovesjö

**Affiliations:** 1 Medical Management Centre, LIME, Karolinska Institutet, Tomtebodavägen 18 A, 171 77 Stockholm, Sweden; 2 Division of Clinical Pharmacology, Department of Laboratory Medicine, Karolinska Institutet at Karolinska University Hospital Huddinge, 141 86 Stockholm, Sweden; 3 Public Healthcare Services Committee, Box 17533, 118 91 Stockholm, Sweden; 4 Stockholm Drug and Therapeutics Committee, Public Healthcare Services Committee, Box 17533, 118 91 Stockholm, Sweden; 5 Clinical Pharmacology, Department of Medicine Solna, Karolinska Institutet, Karolinska University Hospital, 171 76 Stockholm, Solna Sweden; 6 Karolinska Institutet, Department of Public Health Sciences, Tomtebodavägen 18A, Stockholm, Sweden; 7 Department of Clinical Science and Education, Södersjukhuset, Internal Medicine, Karolinska Institutet, 118 83 Stockholm, Sweden

**Keywords:** Drug formulary, Drug and Therapeutic committee, Information sharing, Medicine information, Patient education, Survey interviewing

## Abstract

**Background:**

Involving patients in decisions about their pharmacotherapy is crucial for a satisfactory treatment outcome. Information and opinions about medicines are available from a variety of sources. The Wise List is the drug formulary of recommended essential medicines for the Stockholm healthcare region and is issued by the Drug and Therapeutics Committee (DTC). To inform the public about treatment for common diseases and the concept of recommended medicines, a patient edition of the Wise List was developed. The aim of this study was to explore patients’ knowledge, needs and attitudes to the Wise List, DTC and information about medicines in general.

**Methods:**

To examine patient knowledge about recommended medicines a survey (*n* = 312) was carried out at four large primary healthcare centres in Stockholm, Sweden. To further elucidate the patients’ needs of the information on recommended medicines and medicines in general, three focus group discussions (FGDs) were performed.

**Results:**

Of the respondents 57% did not recognise the Wise List, 26% recognised but did not use it and 17% used it. A total of 63% reported that they search for information about medicines. The most common information source was “asking their doctor” (36%) followed by searching the internet (31%). The FGDs revealed that the patients were not interested in medicines in general, only in the medicines they use themselves. They did not understand the aim of the Wise List or how they could benefit from information about recommended medicines. The patients expressed a wish to access all information they need about their own care as well as public healthcare information at one location.

**Conclusion:**

The intended aim of the DTC with providing information to the public was not achieved as the patients have difficulties to understand the information and how they should use it. The patients were not interested in medicines in general, they wanted information tailored to their specific needs. The findings highlight the importance of creating tools for patients in collaboration with them and evaluate the concept continuously.

## Background

The importance of involving patients in decisions regarding their own healthcare has become increasingly recognised [1, 2]. Pharmacotherapy is a common intervention where patient participation is particularly important since choice of medicine and dosages need to be individualised for an effective and safe therapy [3]. Patients with a comprehensive understanding of their medicines are more likely to ask questions about the medication. When patients ask questions, physicians’ possibilities to correct misunderstandings increase, making treatment decisions more judicious and informed [4].

 Today patients have access to information and opinions about medicines from different sources, including healthcare professionals, pharmacists, manufacturers and discussion forums on the internet. However, to make it possible for patients to participate in treatment decisions, reliable tools and evidence based information are needed. Earlier research has shown that many patients want to participate in the decision making but feel they lack adequate information to do so [1]. Evidence suggests that written information about medicines may influence patients’ knowledge and serve as a counselling tool which further empower patients [5]. However, there is still a lack of knowledge about how patients assimilate information about evidence based medicine and especially treatment guidelines [5]. The significance of health literacy is increasingly acknowledged. There are different definitions in use, but health literacy can be considered to not only depend on the patients’ ability to find, understand and use information to enhance health, but to also reflect the ability of the editors of information to provide comprehensible information for patients [6, 7].

The Wise List is the drug formulary of recommended essential medicines for the entire Stockholm healthcare region. It was developed by the Drug and Therapeutics Committee (DTC) of Stockholm, Sweden in 2000 as a part of the multifaceted approach described as the Stockholm Model for Wise Use of Medicines [8–11]. The Wise List consists of about 200 core medicines and aims to cover first line, and often second line, treatment of common diseases covering several therapeutic areas such as “cardiovascular diseases” and “endocrinology”. The Wise List for prescribers is available in different formats, a pocket-sized booklet and a non-commercial website with a responsive design [12]. To advice patients an adapted version of the Wise List has been developed and are available in a pocket-sized booklet.

The patient version of the Wise List was launched in 2001. The DTC’s aim with the Wise List has been to inform the public about the drug formulary as all information about evidence based prescribing of drugs should be available to patients and public [8]. The Wise List is found in waiting rooms at hospitals and health care centres and at local pharmacies. To raise public awareness of the Wise List, the DTC developed and implemented a communication and marketing strategy focusing both on healthcare staff and patients and the general public as early as 2000–2001. During the first two years following the launch of the Wise List, the DTC carried out campaigns and placed advertisements in newspapers and on public transport in the whole metropolitan region during one month. Thereafter, in connection to the launch of the annual edition, advertisements were placed in the members’ magazines of the senior citizen associations almost every year, and some years also in daily newspapers.

 Earlier studies have shown that physicians believed that patient-adapted information on drug recommendations could increase patient adherence and would be a better alternative to drug advertisement from the pharmaceutical industry [8, 13]. Therefore, the adapted patient version of the Wise List might facilitate shared decision-making about the most appropriate treatment [8]. However, for a patient version of a drug formulary to be useful, members of the public and patients need to be aware of its existence, its contents must be perceived as relevant and the information needs to be understandable [14].

 The aim of this study was to increase our understanding of what patients in Stockholm knew about the Wise List with recommended medicines, how they use it, as well as the kind of information patients would like to have about medicines. The results were intended to be used to further develop the Wise List for patients. To our knowledge this is the first study which explores patients’ views on a drug formulary from a DTC.

## Methods

### Study design

We used a mixed method study design comprising a survey and focus group discussions (FGDs) [15]. This explanatory study design was chosen since the researchers found during the survey data collection, that the respondents had difficulties to express their views about the content in the Wise List and how they used it. Therefore, survey data alone were insufficient to fulfil the aim of the study. To gain a deeper understanding of how patients interpreted the information we decided to complement the survey study with FGDs.

Furthermore, as there is a recognised problem of implementing research findings in practice we were inspired by a learning approach while evaluating the Wise List among patients [16, 17]. The learning approach is based on an increased collaboration between the researchers and the stakeholders in the research process. The purpose of this interactive work is to ensure that the results will be applied in practice. When the stakeholders are involved in the research they are more prone to change the practice according to the results. In the data collection three researchers (MNA, MV and PBR) worked closely with two pharmacists (SE and KAg) who coordinate the Wise List development.

## Survey

### Data collection

With the aim of assessing patients’ knowledge of the concept of the Wise List for patients a survey was undertaken. This survey was conducted using a survey interviewing method where a researcher was physically present to ask the questions and to fill in the respondents’ answers [18]. This approach offers many advantages over mail and telephone surveys in terms of quality of the data collected. If the respondent finds a question unclear the interviewer can immediately clarify it. Similarly, the respondent can be asked to clarify any answers that the interviewer cannot interpret. The survey followed a standardised script encompassing four areas of questions; background variables of the respondents, knowledge about the concept, use of the Wise List and general use of information about medicines. Different responses to the question about knowledge prompted different follow-up questions (Table 1). The survey was pilot-tested on four persons to ensure that the questions were clear and did not compromise the respondents’ integrity.

**Table 1:** Questions to participants’ in the survey

Question area	Questions to *all* participants	Questions to participants *who recognised* the Wise List	Questions to participants *who recognised and use* the Wise List
Background variables of the respondents	Sex Female Male Born year Opened answer Living in the area Yes No		
Knowledge of the concept with the Wise List	Do you recognise the Wise List? Yes No	Do you use the Wise List? Yes No, why don’t you use it? Do you know who produce the Wise List? Yes No	How do you use it? Opened answered Do you found the information you were looking for? Yes No How useful do you found the information in the Wise List? Scale 1–10 (no benefit- great benefit) Would you recommend the Wise List to somebody else? Yes No
Use of general information on medicines	Usually, do you search for information about medicines? Yes No Do you want to have information about medicines? Yes, how? No		

The data collection took place at four large primary healthcare centres (PHCs) in Stockholm, Sweden. The rationale for choosing patients at the PHCs as target population was that at PHCs you meet people with many different diseases who have had to make decisions about their medications, i.e. a place with people with potential experience of, and use for, the drug formulary. The PHCs were selected to achieve a representative sample of Stockholm Healthcare region primary care as to differences in organisation (corporate vs public), geographical location, socioeconomic conditions and number of patients listed at the PHC.

 We decided to collect data 1 day in February 2015. To determine the number of respondents required to fulfil the purpose of the survey, we used information about the number of patients visiting each PHC (in the whole metropolitan area) from the database (called VAL) of Stockholm healthcare region [19]. During the same time-period the year 2014 we could see that the average number of patients visiting the four PHCs in 1 day were 853 (with a range from 177 to 291 patients for each PHC, see Table 2). Two of the selected PHCs are located in parts of the Stockholm region with a higher socioeconomic status (south east and north east) and two of them are located in less affluent parts (northwest and south). We assumed that the knowledge about the Wise List would be higher in the former (60%) as compared to the latter (30%). In each PHC, we decided to interview 35% of the expected daily number of visiting patients. The equal proportion was chosen to ensure that our sample population would be representative of the total population in these four PHCs. A selection of 35% would, under these assumptions, give us a standard error of approximately 0.029 for the estimated total proportion, which we deemed satisfactory.

 At the PHCs the researchers asked adult patients, who entered or exited the waiting room, if they wanted to participate in the survey. The researchers read out the questions and filled in the respondents’ answers. Each survey lasted 5 to15 minutes. In the cases where the respondent did not speak Swedish (*n* = 2), the questions were translated into English. The data were collected by four of the co-authors (KAg, MNA, SE and PBR). We collected 312 responses (Table 2). Around 10 patients declined to participate at each PHC.

**Table 2:** Number of selected respondents per primary healthcare centres

Primary healthcare centres organisational setting	Geographic location in Stockholm	Average number of visits per day in week 6, year 2014	Number of surveys to be collected (35% of all visits) and collected surveys (n)
Corporate	South	177	63 (66)
Public	South East	291	102 (105)
Public	North West	195	69 (71)
Corporate	North East	190	66 (70)
Total		853	300 (312)

### Data management and analysis

All survey data were manually entered in Microsoft Excel 2010. The data were then summarised into numbers and percentages and are shown in tables and figures.

## Focus group discussions

### Data collection

FGDs were chosen as data collection method since it is a particularly valuable method in examining how people think about an unexplored topic [20]. In FGDs, in contrast to in individual interviews, participants hear each other’s responses and can give additional comments and expand their answers [20]. In order to capture a wide range of experiences, a strategic sampling was performed including patients with a large variety of conditions and diagnoses treated with different medications.

In total, three FGDs were performed with 13 participants (10 women) between 40 and 70 years old. There were 4–5 patients participating in each FGD. All FGDs took place at the Healthcare Administrations office in Stockholm, April 2015. One FGD consisted of patients from different PHCs and the other two with patients from two patient organisations; DHR, an organization for people with impaired mobility [21] and HSO, the Swedish Disability Federation [22]. Participants were recruited through contact persons for the patient organisations and by contacts from the PHC. When a person accepted to participate in a FGD a copy of the Wise List for patients was sent to the participant and the participant was asked to read it prior to the FGD.

A semi-structured discussion guide was used (Table 3) and each FGD lasted 1.5 h. All discussions were audio tape recorded and transcribed verbatim. One researcher acted as moderator, another as observer and a pharmacist was present to answer questions from the participants about the Wise List.

**Table 3:** Semi-structured discussion guide with topics used in the FGDs

Introductory question about medicines in general	
- Do you usually search for information about medicines? - How do you search for information? - What kind of information do you want about medicines?	
Use and usefulness of the list with recommended medicines, i.e. the Wise List	
- What is your first impression of the Wise List? Have you used it before? If, how does it worked? - How do you use the Wise List? Can you give an example of a situation? - Is it easy/difficult to find the information you are looking for? What is difficult? - Is there anything in the Wise List that you think are particularly good/especially bad? - What do you think about the content in the Wise List? - How do you perceive the purpose of the Wise List? - How would you improve the Wise List?	
Concluding questions	
- After a brief summary - Is there anything you want to add? - How did you find it to discuss this topic?	

### Data analysis

An inductive thematic analysis with no predetermined categories were performed in a stepwise manner [16], see Table 4. In this approach, the categories identified are strongly linked to the data themselves without trying to fit into a pre-existing theoretical frame [23]. The analysis was conducted by two researchers with extensive experience in qualitative methodology (MV and PBR). The results were then validated by two co-authors (SE and KAg) who had participated in the data collection and read all transcripts. The main aim of this process was to compare the results to determine whether any categories was overlooked. The four authors then took part in the analysis process of finding related patterns between the emerging categories through a reciprocal reading between the transcribed text and the categories. This is a way to increase the trustworthiness in the analysis process [24].

 Data saturation usually means that data should be collected until no additional patterns or themes/categories emerge from the data [25]. In the analysis of the third FGDs no more categories emerged from the data related to the aim of the study, thus saturation was assumed.

**Table 4:** Description of the thematic analysis process

1.	Tapes and transcripts from the FGDs were listened to and read repeatedly to get a good grasp of the material.
2.	Sections of text in the transcripts, focusing on the research question, were marked. Marked sections with related topics were grouped into emerging categories: 1) use of recommended medicines (the Wise List), 2) understandings of the aim, 3) benefits and needs, 3) improvements, and 4) general information about medicines.
3.	The sections of text in each category were summarised and grouped by content into preliminary subcategories.
4.	The next step was to find related patterns within each preliminary subcategory. Sections of text were moved between subcategories and new subcategories were formed.
5.	After negotiated consensus between the researchers, the subcategories were grouped into three categories with related sub-categories (Table 5).
6.	Quotes were chosen to illustrate and validate each sub-category.

### Ethical considerations

This project was part of on-going quality improvement work at Stockholm healthcare region.

All handling in this study is complied with Swedish legislation [26] and no application for approval by the Regional Institutional Review Board was needed. In the survey’s respondents were informed about the aim of the study and thereafter asked if they wanted to participate. No personalised data about the respondents were collected. In the FGDs all participants were informed of the purpose of the study. After receiving written and verbal information, all respondents gave their written consent to participate. Respondents were assured that their participation was confidential and voluntary, and that they had the opportunity to withdraw at any time. The interviews were audio-taped after approval by the respondents. The identities of the respondents were removed from the transcripts to guarantee confidentiality. Furthermore, to guarantee respondents to be anonymous the citations in the results is neither labelled with name nor which FGD the citation is derived from.

## Results

### Results from the survey

#### Background information respondents

Three hundred twelve respondents answered the survey, 59% were women. The mean age was 56 years, with interquartile range 18–96 years. Most of the respondents (89%) lived in the area where they answered the survey.

#### Reported knowledge and use of the wise list

**Fig. 1:**
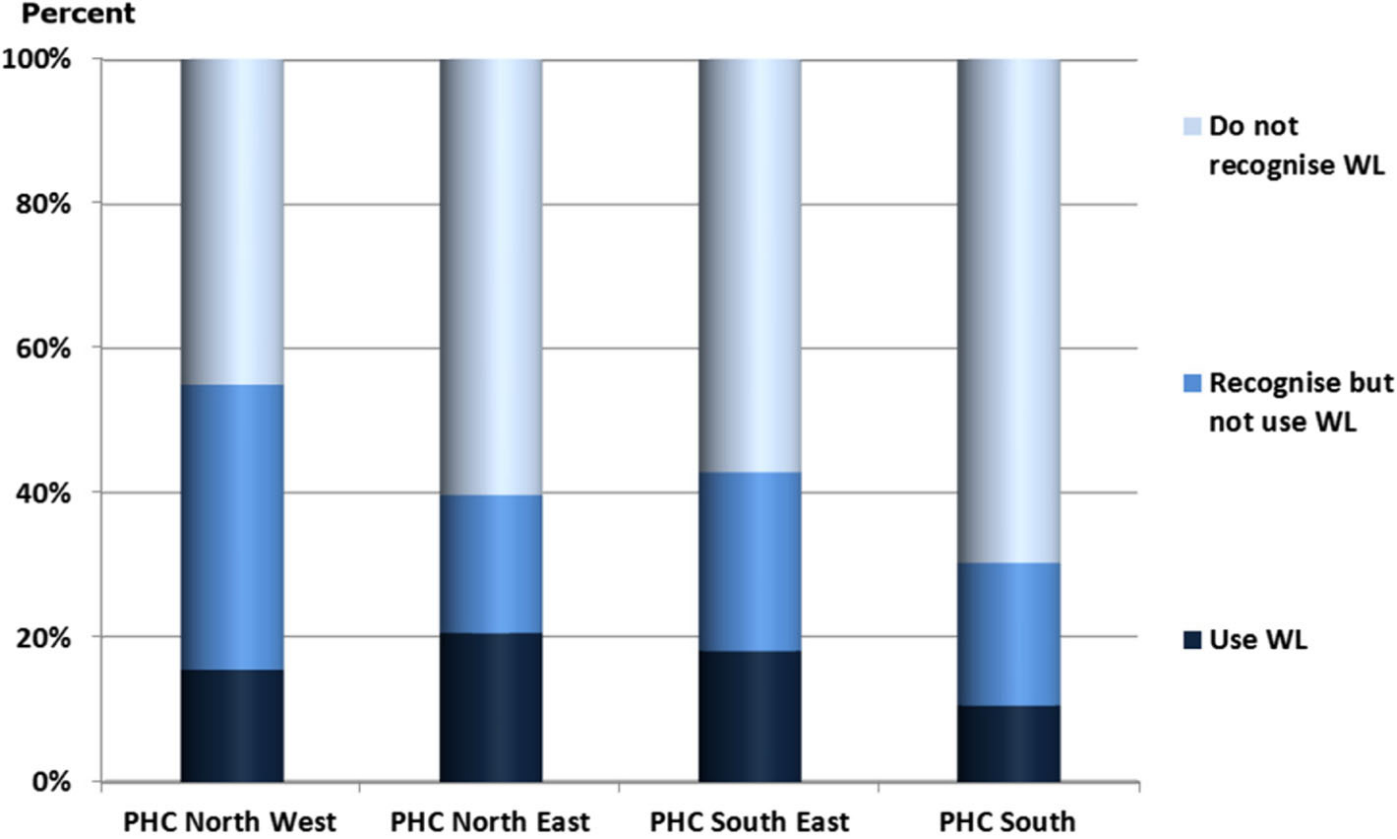
Comparison between the four Primary Healthcare Centres regarding knowledge and use of recommended medicines (the Wise List) and Primary Healthcare Centre (PHD)

**Fig. 2:**
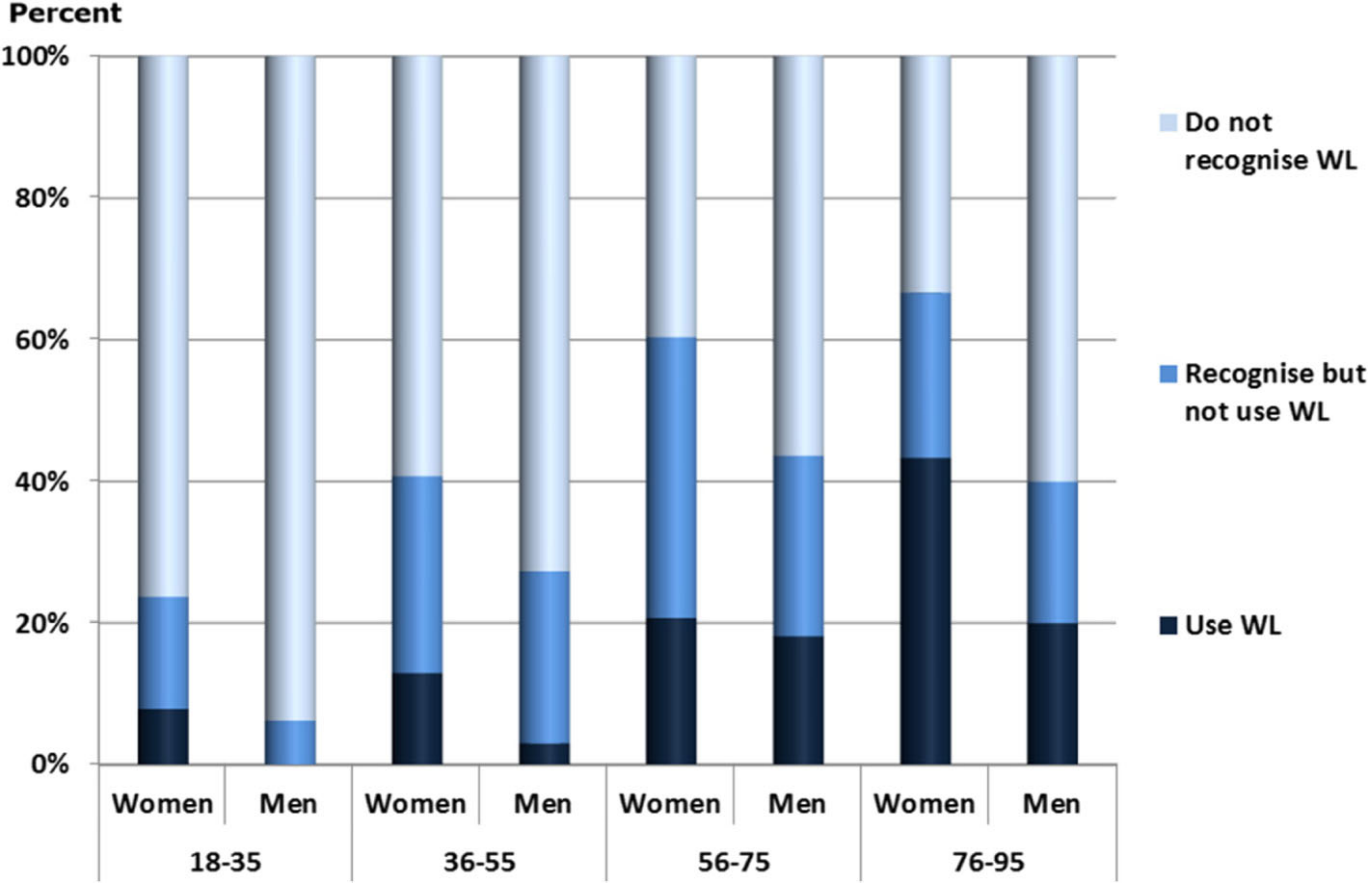
Comparison between age and knowledge and use of recommended medicines (the Wise List)

Of the respondents 57% did not recognised the Wise List, 26% recognised it but did not use it and 17% recognised and used it. Comparing the four PHCs a tendency could be seen towards the northern districts in Stockholm Healthcare region having a slightly higher knowledge and use of the Wise List (Fig. 1). The use of the Wise List was higher among women than men in all age groups and higher among elderly than among younger people (Fig. 2).

 Of the Wise List users (*n* = 53), 34% answered that they use the Wise List when they have a question about medicines, and 32% that they have just flicked through it. 66% said that they find the information they are looking for. One in four (24%) reported that they benefit greatly from the information in the Wise List. The majority of the users (81%) said they would recommend the Wise List to someone else. Of all 53 respondents who recognised and used the Wise List, a minority (16%) knew that the DTC in the Stockholm healthcare region is the sender of the Wise List.

#### Reported general use of drug information

Of the 312 respondents, 63% reported that they search for information about medicines. The most common information source was “asking their doctor” (36%) followed by searching the Internet (31%). In contrast, only 37% stated that they want to have information about medicines.

### Results from the focus groups discussion

During data analysis, we identified three categories with underlying sub-categories (Table 5).

**Table 5:** Categories and sub-categories of respondents understanding with the concept of recommended medicines (the Wise List), emerging from the analysis of the FGDs

Category	Sub-category
Knowledge, attitudes and use of the Wise List	✓ Knowledge of the Wise List ✓ Unclear aim ✓ The content is on a too high theoretical level ✓ The risk of misunderstandings and misuse of medicines
Suggestions for improvement	✓ A simplified list of recommended medicines ✓ Make all information about medicines available in one place and in “print on demand machines”
Medicine information in general	✓ Individualised information about medicines ✓ Sources of information about medicines and reliable producer of information

#### Knowledge, attitudes and use of the wise list

##### Knowledge of the wise list

Before participating in the FGDs many respondents had heard about the Wise List. However, few had read it and they did not know what it contains more than it had something to do with *“healthcare or medicines”*. Some respondents had never heard about the Wise List before and were surprised that they had never received information about it.

*I’ve never heard of it or seen it! And it’s been around for fourteen years. I was really surprised. I mean…where has it been?*

##### Unclear aim

Common for all respondents was that they had not understood the aim of the Wise List, or how to use the information in it. Some had tried to use it but failed.

*I don’t really understand how you are meant to read this table. It’s really simple…but I still can’t understand it.*

Few had understood that the information was about recommended medicines for common diseases. Furthermore, they did not understand the concept of recommended medicine. After clarification of the aim from the pharmacist, some respondents questioned if this kind of information was useful for patients.

*Is the idea that everyone should accept the procured medication? I mean, I don’t really understand what it’s for. I can understand why doctors have it...But are we supposed to understand why doctors choose certain medicines? I mean…is that what it’s for?*

The respondents felt that the sender’s message did not reach the intended users of the information. During the discussion, the respondents asked questions like: *“What is the benefit with information about recommended medicines for patients?”* and *“How does the sender want the patients to use the information?”* The respondents emphasised that the aim, with instruction to patients of how to use it, should be clearly written in the beginning of the Wise List, otherwise they would not benefit from the information.

*I think it should clearly state why it is produced and how I, the patient can benefit from it. Perhaps I will understand if I read it again but I mean how many people read it again?*

##### The content is on a too high theoretical level

The respondents’ found the information in the Wise List is difficult to understand and that it is on a *“too high theoretical level”* for the general patient. The information includes medical terms that are incomprehensible for a person not trained in healthcare. Additionally, the respondents described the text as confusing without a common structure. They also thought that it is difficult to understand the context of the text.

*I’m an experienced reader and when I get lost reading it then there must be something wrong with the set-up.*

One respondent had gone to the pharmacy to fill a prescription for a branded medicine and the pharmacist suggested the generic equivalent instead. When the respondent read about the medicine in the Wise List she found that she did not understand the information and was therefore not helped by the list.

*I don’t get it and yet it’s written for patients. I mean we don’t understand this about active substance, we don’t understand what is medicine and what is active substance…but somehow, I want to believe that the active substance is just as strong or potent regardless of which tablet it is, but I’m not sure whether I can believe that or not and then I didn’t understand what I should compare with...*

##### The risk of misunderstandings and misuse of medicines

The respondents did not know how to interpret the information in the Wise List. Some found it difficult to understand the difference between first- or second line treatment. One respondent perceived the information as a kind of self-care advice where the patient should start with the substance recommended as first line therapy and if it is not sufficient go on to the second choice and so on.

*…I have that diagnosis…and then when I get a migraine…should I take a Pamol first, which is the first choice, and then take a Magnecyl as well? Should I take both or one of each? If it doesn’t go away, then I should take the second choice…if it doesn’t help…then I wonder how long I should wait before I take the second-choice medication?*

#### Suggestions for improvement

##### A simplified list of recommended medicines

One of the respondents’ suggestions was to make the Wise List patient version a folder of three to four pages with information about common medical conditions and a list of the recommended medicines. Another suggestion was to only have a list of recommended medicines. The information should be written in simple language without medical terms, and with understandable symbols.

The respondents thought that it should be clearly stated that the medicines are recommended by experts. Knowing that a treatment is recommended by an expert can create a feeling of security *“to know that the medicine treatment is evaluated by an expert inspires trust”.* They also thought that is should be made clear that a physician may prescribe medicines that are not on the Wise List, and that it does not mean there is something wrong with the prescription. *“If I can’t find my medicines on the Wise List, then I don’t need to think that my doctor has made a mistake”.*

##### Make all information about medicines available in one place and in” print on demand machines”

The respondents had different views on where and how the information should be available. Some of them wanted printed material, some an app for mobile phones but most respondents wanted to have the information available on the Internet. Furthermore, they thought it would be good to collect all healthcare information directed to patients in one place. As an example they mentioned the website 1177 Vårdguiden. This is a website where patients can find information about their own care as well as public information about the healthcare system [27]. Another suggestion was the establishment of “print on demand machines” in the PHCs where the patient or physician can print out information on a specific medicine like. *“the machine in the supermarket where you can push a button and get your cooking recipes”.*

#### Medicine information in general

##### Individualised information about medicines

Many respondents wanted to have information targeted to them, because they wanted to be involved in their own treatment. However, the respondents expressed that they were not interested in medicines in general, they just wanted information on the medicine they are using. There were also differences in how well informed the respondents wanted to be and due to this their information searching behaviour varied. Some respondents did not seek information on medicines because they *“rely on the doctor’s prescription”*, while others say that they are searching for more specific information about medicines than they have received from the physician. Examples of information the respondents searched for were: what impact and effect a medicine has on the body, side effects, drug interactions, generic replacement, medicine packaging and how the medicine works in combination with alcohol or driving.

*First, you want to know if it helps for the symptom or illness you have. Then you want to know that it won’t harm you in some way, somewhere in your body.*

##### Sources of information about medicines and reliable producers of information

The most common source of information about medicines was the physician. Nevertheless, many respondents double check their medicines by seeking additional information, especially regarding adverse effects. The respondents reported to seek information mainly on the Internet.

Many searched randomly, without a specific source in mind *“just to see what I get”*, while others seek more familiar sources with reliable senders like Physicians Desk References or the public healthcare website 1177 Vårdguiden [27], and some read the package insert. The respondents had different perceptions about the importance of the source of the information. Some thought it was important with a reliable sender, while others said that the source does not matter.

*We want easily understood and correct material because there is a lot of rubbish on the internet. You have to have sources that are trustworthy and this (The Wise List) is, so that’s important.*

## Discussion

This study demonstrates that a large proportion of patients (43%) know about recommended medicines in the Wise List for patients. The knowledge was slightly higher among elderly people and women, which is not surprising as they use more medicines than younger people and men [28]. However, among the respondents who reported that they have knowledge about the Wise List only 17% reported using it.

There seem to be several reasons for the low use of the Wise List for patients. According to the findings from the FGDs, the respondents did not understand the aim of the Wise List or how they could benefit from information about recommended medicines. Furthermore, the respondents did not understand how to interpret the information in the Wise List. Some reported it difficult to understand what it means when a medicine is specified as first- or second line treatment. Also, there are medical terms and expressions that are incomprehensible for a lay person. In the FGDs, the respondents started to ask questions to the research group about the sender’s intention/aim with the Wise List and how they could benefit from reading it. This is an interesting result showing the necessity of clarifying the aim of products like the Wise List as well as simplifying the contents in order for patients to make use of the information in a safe way.

A surprisingly high proportion (63%) of the respondents stated that they did not want information about medicines. In the FGDs, the respondents expressed that they did not feel a need for general information about medicines, only for information about the medicines they use. This view is in concordance with other studies where patients report that information in guidelines often are too general and that they want information that is specific to them [14, 29].

The Wise List for patients was an attempt to adapt the information for physicians to patients in general, which resulted in a small amount of information about the treatment of many conditions. This study demonstrates the need for a much more individualised approach when providing information to patients. Hamrosi et al. also found that the patients primarily discuss their concerns with their physician who is able to discuss the treatment, not in general terms but from the patient’s specific situation [5].

In our study patients thought it would be useful to know that the recommended medicines have been selected by experts, a view recognised from the literature [14]. In addition to discussing with their physician, many respondents seek verification of the appropriateness of the treatment on the internet and a suggestion from the respondents was to collect all information to patients in one place where patients can find information about their own care as well as public healthcare information.

There are a number of models of shared decision making between patients and prescribers, but a common feature is that patients must have access to different kinds of support tools to be able to participate in decisions about their own treatment [1, 2]. One significant barrier that prevents shared decision making is that patients lack adequate information [1]. The Stockholm DTC’s aim with the Wise List for patients is to make information about recommended medicines available to patients and public, i.e. patients should have access to the same information on evidence based medicine as physicians to enable involvement in care. The intended outcome was not achieved as the patients have difficulties to understand the information provided and how to use it. This study elucidates the importance of not creating tools *for* patients, but to do it *with* patients.

The Wise List for patients has now been revised according to the input from this study and are presented on the website 1177 Vårdguiden [30] with the most important information being presented first and options for the users to access more detailed information if needed [14]. The printed version has also been simplified. Evaluations of these changes will be made in collaboration with patient organisations. Including co-workers who participate in the editorial work of the Wise List for patients in the study, may have contributed to the readiness of the DTC to act on the results.

### Limitations

All survey data were collected in the context of primary care by interviewing adult patients entering or exiting the waiting room of a PHC. This method may affect the selection of patients, since there was only one interviewer per PHC and not all consecutive patients could be interviewed. Patients participating in the survey ranged between 18 and 96 years and 59% were women.

The fact that the survey data were collected during 1 day, a Tuesday, could have led to bias on the interviewed participants if the patients who visit the PHC different days have different knowledge about the Wise List. We chose to collect the survey data in 1 day because of practical constraints. Also we did not expect significant differences in the characteristics of patients between different days of the week.

One limitation could be that we performed the survey only in four PHCs (out of approximately 200 PHCs in the Stockholm Healthcare region). But by selecting PHCs with differing organisation, geographical location and socioeconomic conditions in the catchment area we deem that the findings are valid for the general population of Stockholm. Also, the differences in knowledge about the Wise List between PHCs from different socioeconomic areas were smaller than we had assumed, ranging from 30% (the PHC south) to 55% (the PHC north west). However, with both quantitative (survey) and qualitative (FGDs) findings we consider that the results are applicable to similar contexts and settings when conveying evidence based non-commercial information about medicines to patients.

## Conclusion

A high proportion of patients recognised the patient edition of the drug formulary, called the Wise List for patients. However, the DTC’s aim with the Wise List was not achieved as the patients had difficulties understanding the information about recommended medicines and how to use it. The patients were not interested in medicines in general, they wanted information tailored to their specific treatment. The respondents expressed a wish to find information about their own healthcare as well public healthcare information in one place. To enable patients to participate in decision making about pharmacotherapy, they need information that they perceive as relevant. If the DTC wants to communicate a message about medicines to patients, patients need to be more involved in the process. The findings highlight the importance of not creating tools *for* patients but to do so *with* patients and evaluate the concept continuously. An approach to facilitate implementation of research findings into practice is to include the stakeholders in the research process.

## Abbreviations

DTC: Drug and Therapeutics Committee
FGDs: Focus Group Discussions
PHD: Primary Healthcare Centre
